# The effect of informal caregiver support on utilization of acute health services among home care clients: a prospective observational study

**DOI:** 10.1186/s12913-018-2880-9

**Published:** 2018-01-31

**Authors:** Robert Abtan, Nooshin Khobzi Rotondi, Alison Macpherson, Michael Anthony Rotondi

**Affiliations:** 1 School of Kinesiology and Health Science, Faculty of Health, York University, Toronto, ON Canada; 2 Musculoskeletal Health & Outcomes Research, Li Ka Shing Knowledge Institute, St. Michael’s Hospital, Toronto, ON Canada

**Keywords:** Emergency department visits, Hospitalizations, Community care, Informal caregivers

## Abstract

**Background:**

Emergency department visits and hospitalizations (EDVH) place a large burden on patients and the health care system. The presence of informal caregivers may be beneficial for reducing EDVH among patients with specific diagnoses. Our objective was to determine whether the presence of an informal caregiver was associated with the occurrence of an EDVH among clients 50 years of age or older.

**Methods:**

Using a database accessed through the Toronto Central Community Care Access Centre (CCAC), we identified 479 adults over 50 years of age who received home care in Toronto, Canada. Exposure variables were extracted from the interRAI health assessment form completed at the time of admission to the CCAC. EDVH data were linked to provincial records through the CCAC database. Data on emergency room visits were included for up to 6 months after time of admission to home care. Multiple logistic regression analysis was used to identify factors associated with the occurrence of an EDVH.

**Results:**

Approximately half of all clients had an EDVH within 180 days of admission to CCAC home care. No significant association was found between the presence of an informal caregiver and the occurrence of an EDVH. Significant factors associated with an EDVH included: Participants having a poor perception of their health (adjusted OR = 1.68, 95% CI: 1.11–2.56), severe cardiac disorders (adjusted OR = 1.54, 95% CI: 1.04–2.29), and pulmonary diseases (adjusted OR = 1.99, 95% CI: 1.16–3.47).

**Conclusions:**

The presence of an informal caregiver was not significantly associated with the occurrence of an EDVH. Future research should examine the potential associations between length of hospital stay or quality of life and the presence of an informal caregiver. In general, our work contributes to a growing body of literature that is increasingly concerned with the health of our aging population, and more specifically, health service use by elderly patients, which may have implications for health care providers.

## Background

The use of acute care services, such as emergency department visits and hospitalizations (EDVH) is costly, limits continuity of care and adds a substantial burden to the health care system [1]. In the 2011–2012 fiscal year, costs of emergency room visits in Ontario, Canada, were $1.1 billion [2]. According to the Canadian Institute for Health Information [3], in 2012–2013, the cost of acute inpatient hospitalizations in Canada was $24.4 billion. Moreover, recent work in Ontario has shown that frequent EDVH are often considered indicators of poor quality of end-of-life care [4, 5].

The presence of informal caregivers has shown conflicting results with respect to reducing EDVH. The term informal caregiver refers to an unpaid individual, such as a family member or a friend, who is involved in assisting others with activities of daily living and/or medical tasks [6]. In patients with cardiac disease, having an informal caregiver may be beneficial for reducing EDVH [1]. However, in a broad study of patients 18+ years of age, Weaver & Weaver [7] found that the availability of an informal caregiver in the household did not affect hospitalization rates among study participants. Van Houtven and Norton [8] also reported that informal care provided by adult children did not significantly affect the likelihood of hospitalization for single individuals aged 70 and older. A subsequent study by the same authors [9], which examined Medicare expenditures among single elderly patients, found that informal care by adult children did not affect the occurrence of a Medicare-financed hospitalization, but did reduce Medicare expenditures. Saunders [10] found conflicting results showing that both the lack of family support and increased caregiver hours at home were associated with increased hospitalizations among patients with heart failure. Using the 2004 Survey of Health, Ageing and Retirement in Europe, Bolin et al. [11] found that informal care provided by children and grandchildren (the mean age of the eldest child was 37 years) increased the likelihood of hospitalizations in single individuals aged 50 and older. Finally, in a cohort of 55 to 85 year old adults from the Longitudinal Ageing Study Amsterdam, Pot et al. [12] found that living with a partner increased the likelihood of an individual to see a medical specialist, but showed no significant association with the occurrence of a hospitalization.

Given the variety of conflicting evidence, additional research in this area is warranted. If the presence of informal caregivers is found to be beneficial with respect to reducing EDVH, support for caregivers may indirectly help to alleviate the financial strain on the health care system, and assist in care planning [4]. Conversely, the absence of informal caregivers may be used to identify individuals who require additional support. Thus informal caregivers may be instrumental in increasing quality of life for patients prone to more frequent use of acute care services [1, 4, 13].

The primary objective of this study was to determine whether the presence of an informal caregiver was associated with the occurrence of an EDVH among patients receiving home care through the Toronto Central Community Care Access Centre (CCAC). To examine this association, data were obtained from the CCAC, whose responsibilities included assessment, management, and delivery of home care services to individuals with the goal of keeping them at home, and mitigating the use of acute care services such as hospitalization and long-term care [4, 14].

## Methods

### Study population

The sample was obtained from a database of 479 adults 50+ years of age who had been admitted to St. Joseph’s Health Centre, a community teaching hospital in Toronto, Canada. These patients were subsequently admitted to the Toronto Central CCAC from January 1, 2012 to August 1, 2014. Only patients with a completed interRAI (International Resident Assessment Instrument) [15], a health assessment tool, around the time of admission to the CCAC were included in the analysis. Data on emergency room visits were included for up to 6 months after time of admission. Participants who did not have complete data for the 6-month time period (e.g. died prematurely), were included in the analysis to increase external validity and the representativeness of the study population. Ethics approval was obtained from St. Joseph’s Health Centre and the Toronto Central CCAC. No personally identifying data were used for this analysis.

### Data sources

Exposure variables were obtained from the interRAI assessment form. The interRAI is a standardized health assessment tool that evaluates patients’ health care needs, demographics, health conditions, and other related variables [16]. The version of the interRAI used for this study was the Minimum Data set – Home Care (MDS-HC), which applies specifically to home care [17]. It includes questions on health conditions, service utilization, behaviour, physical and cognitive functioning, and demographic information, such as age, gender and smoking and drinking habits. The reliability of the interRAI has been well established [4, 16].

Some participants had multiple interRAI assessments; however, we used the most recent interRAI, which could have been completed up to 30 days subsequent to admission to home care. The rationale behind this decision was to include participants who had not completed an interRAI until after admission. The interRAI data were linked to EDVH data via the use of anonymized CCAC client numbers.

Data on the outcome variable (EDVH) were collected from the National Ambulatory Care Reporting System (NACRS) and the Discharge Abstract Database (DAD), which were recorded by the Canadian Institute for Health Information and linked to interRAI data using anonymized CCAC client numbers. NACRS contains information on the occurrence of emergency room visits, while DAD includes acute hospital admissions [4, 18]. For reference, all exposure and outcome variables were made available to the research team in a complete and de-identified database by the Toronto Central CCAC.

### Exposure variables

The primary exposure variable, “Live-in Caregiver Support,” was selected based on its use in previous studies analyzing the utilization of acute care services [1, 7]. The variable was binary and categorized as “Presence of an informal caregiver” and “Absence of an informal caregiver”. “Live-in Caregiver Support” was obtained from Section G - “Informal Support Services” of the interRAI.

Other variables of interest included: age (in years), gender, marital status (married or not), English as the primary language, education level, individual disease diagnoses, pain frequency, drinking (within the last 90 days from completing the interRAI, participant felt the need or was told by others to cut down on drinking, or others were concerned with the participant’s drinking), smoking (smoked or chewed tobacco daily), participants’ perception of health (if client felt they had poor health), and Activities of Daily Living decline (if ADL status had become worse in the past 90 days with respect to the time of interRAI completion or since the last interRAI assessment). Pain frequency and education level were categorical variables, while all other covariates were binary.

Self-reported disease diagnoses included those that were present at the time of completion of the interRAI, were monitored by a home care professional, or had been the reason for a hospitalization in the last 90 days since completion of the interRAI. Certain diagnoses were grouped for this analysis. Severe Cardiac Disorders included any case of cerebrovascular accident (stroke), congestive heart failure, or coronary artery disease. These conditions were considered severe as each contributes heavily to mortality and can often require rapid emergent treatment [19–24]. Non-Severe Cardiac Disorders included cases of hypertension, irregular pulse, and peripheral vascular disease. Fractures included hip fractures and other fractures. Senses included cases of cataracts and glaucoma [25]. Pulmonary diseases included cases of emphysema, chronic obstructive pulmonary disorder, or asthma [26]. Urinary tract infection included only cases that had occurred within 30 days of completing the interRAI. Head trauma and HIV were excluded due to a low prevalence (frequencies less than five) of each in the study sample.

### Outcome variable

The primary outcome variable was the occurrence of an emergency room visit or hospitalization within 180 days (approximately 6 months) of admission to CCAC home care. This binary variable was referred to as EDVH, and a positive outcome corresponded to the occurrence of at least one hospitalization, emergency room visit, or both within the study-time period. The occurrence of an emergency room visit or hospitalization could not be analyzed separately, as there was too much overlap between the variables. For example, in terms of DAD, only five of 174 hospitalizations were not accompanied by an emergency room visit.

### Data analysis

Statistical analysis was performed using R statistical software 3.10 [27]. Logistic regression models were used to analyze the data and yielded results in terms of odds ratios (OR) and 95% confidence intervals (CI). The association between each variable and the occurrence of an EDVH was initially analyzed at the bivariate level using simple logistic regression. Following this, we performed an adjusted multiple logistic regression model, which included variables based upon their significance at the bivariate level and their relevance in the literature.

## Results

### Sample characteristics

Of the 479 participants included in the analysis, 215 (44.9%) indicated the presence of an informal caregiver (Table 1). Among the participants who reported an informal caregiver, 114 (53.0%) had an EDVH within 6 months of admission to the CCAC. In contrast, 126 participants (47.7%) had an EDVH among the 264 individuals who did not indicate the presence of an informal caregiver. The average age of participants in the groups with and without an informal caregiver was very similar (82.7 years vs. 82.0 years, respectively), and ranged from 50 to 105 years of age. Only 10.2% of the individuals without an informal caregiver were married, in contrast to 54.9% of the group with an informal caregiver. Education level was missing or unknown for 34.2% of the individuals in the sample.

**Table 1:** Baseline characteristics of Toronto Central CCAC clients, with and without an informal caregiver

Variable	Presence of an Informal Caregiver (*n* = 215), n (%)	Absence of an informal Caregiver (*n* = 264), n (%)
Use of Acute Care Services		
Occurrence of an EDVH	114 (53.0%)	126 (47.7%)
Occurrence of an Emergency room visit	111 (51.6%)	124 (47.0%)
Occurrence of a Hospitalization	85 (39.5%)	89 (33.7%)
Age (years), mean (STD)	82.7 (10.5)	82.0 (11.5)
Gender		
Male	89 (41.4%)	83 (31.4%)
Female	126 (58.6%)	181 (68.6%)
Marital Status		
Married	118 (54.9%)	27 (10.2%)
Not Married	97 (45.1%)	237 (89.8%)
English as the Primary Language		
Yes	98 (45.6%)	151 (57.2%)
No	117 (54.4%)	113 (42.8%)
Education Level		
Bachelor or Higher	15 (7.0%)	18 (6.8%)
High School	26 (12.1%)	33 (12.5%)
Less than High School	79 (36.7%)	81 (30.7%)
Some Post-Secondary or Technical trade school	24 (11.2%)	39 (14.8%)
Unknown	71 (33.0%)	93 (35.2%)

### Bivariate analysis

Several variables were positively associated with the occurrence of EDVH at the bivariate level (Table 2), including Severe Cardiac Disorders (OR = 1.62, 95% CI: 1.12–2.37), Pulmonary Diseases (OR = 2.12, 95% CI: 1.26–3.66), and Poor Perception of Health (OR = 1.80, 95% CI: 1.21–2.70). There were no significant effects among the other covariates, including the primary exposure variable, “Live-in Caregiver Support” (OR = 1.24, 95% CI: 0.86–1.77).

**Table 2:** Bivariate analysis of the association between exposure variables and EDVH within 180 days of admission to Toronto Central CCAC home care

	Unadjusted OR (95% CI)
Main exposure variable of interest	
Presence of an Informal Caregiver	1.24 (0.86–1.77)
Demographics and General Health Variables	
Age	0.99 (0.97–1.01)
Gender (female vs. male)	0.78 (0.54–1.13)
Marital status (married vs. not married)	1.10 (0.74–1.62)
English as primary language	0.88 (0.62–1.26)
Participant or others around them concerned with participant’s drinking habits	0.71 (0.21–2.24)
Smoked or chewed tobacco	1.19 (0.61–2.35)
Felt they had poor health	1.80 (1.21–2.70)*
Experienced ADL decline in last 90 days	1.41 (0.94–2.11)
Education Level	
Bachelor or Higher	0.83 (0.39–1.77)
High School	1.36 (0.75–2.50)
Some Post-secondary or Technical trade school	1.33 (0.74–2.41)
Less than high school	1.00
Pain Frequency	
Daily-multiple periods	1.00 (0.66–1.52)
Daily-one period	1.56 (0.80–3.10)
Less than daily	1.01 (0.56–1.80)
No Pain	1.00
Disease Diagnosis	
Severe Cardiac Disorders	1.62 (1.12–2.37)*
Non-Severe Cardiac Disorders	0.97 (0.67–1.41)
Alzheimer’s or Dementia	0.80 (0.52–1.20)
Hemiplegia/hemiparesis, Multiple Sclerosis, or Parkinsonism	0.89 (0.35–2.25)
Arthritis	0.79 (0.55–1.14)
Fractures	0.94 (0.58–1.51)
Osteoporosis	0.90 (0.59–1.39)
Senses	0.82 (0.53–1.26)
Any Psychiatric Diagnosis	1.20 (0.70–2.04)
Pneumonia or Tuberculosis	1.61 (0.53–5.41)
Urinary Tract Infection (UTI) in last 30 days	0.84 (0.36–1.91)
Cancer	1.46 (0.82–2.64)
Diabetes	1.28 (0.86–1.93)
Pulmonary diseases	2.12 (1.26–3.66)*
Renal Failure	1.59 (0.54–1.13)
Thyroid Disease (hypo or hyper)	0.71 (0.43–1.15)

*p < .05

### Multivariable analysis

A multiple logistic regression model (Table 3) was used to investigate the adjusted effect of Live-in Caregiver Support on the occurrence of an EDVH. The following covariates were included in the multivariable model based on their relevance in the literature despite their non-significance in the bivariate analysis: age, gender, and ADL decline (Table 3). Significant predictors of EDVH in the final model were Poor Perception of Health (OR = 1.68, 95% CI: 1.11–2.56), Severe Cardiac Disorders (OR = 1.54, 95% CI: 1.04–2.29), and Pulmonary Diseases (OR = 1.99, 95% CI: 1.16–3.47). There were no significant effects among the other covariates, including the main exposure variable of interest: presence of an informal caregiver.

**Table 3:** Multiple logistic regression model of the association between the presence of an informal caregiver and EDVH within 180 days of admission to Toronto Central CCAC home care

Variable	Adjusted OR (95% CI)
Presence of an Informal Caregiver	1.16 (0.80–1.69)
Age	0.99 (0.97–1.01)
Gender (female vs. male)	0.82 (0.56–1.22)
Felt they had poor health	1.68 (1.11–2.56)*
Experienced ADL decline in last 90 days	1.33 (0.88–2.04)
Severe cardiac disorders	1.54 (1.04–2.29)*
Pulmonary diseases	1.99 (1.16–3.47)*

*p < .05

## Discussion

Approximately half of the adults in this study had an EDVH in the follow-up period. This did not differ significantly by the presence or absence of informal caregiver support, even after adjusting for other relevant covariates in the model, such as age, gender and disease diagnoses. However, there were significant positive associations between the occurrence of an EDVH and poor perception of health, severe cardiac disorders, and pulmonary diseases.

The lack of association between informal caregiver support and EDVH was consistent with a previous study by Weaver & Weaver [7], where the availability of an informal caregiver in the household did not affect hospitalization rates among inpatients aged 18+ years of age. On the other hand, Schonwetter et al. [1] found that the presence of informal caregivers was beneficial for reducing EDVH. However, this may have been due to the study sample exclusively including patients with cardiac disease. It should be noted that in the study by Weaver & Weaver [7] and Van Houtven and Norton [8], the availability of informal care had an effect on reducing length of stay for hospitalizations, suggesting that informal care may still play a role in terms of quality of life and use of acute care resources. In contrast, the study by Bolin et al. [11] found no association between length of hospital stay and informal care. As a further note, Garrison et al. [28] noted that elderly Medicare patients who were married were at a decreased risk of 30-day readmission. This was echoed in a systematic review by Garcia-Perez [29] in which one of the studies found that widowhood was a risk factor for readmission [30].

Severe cardiac disorders and pulmonary diseases appeared to be a driving factor behind the increased use of EDVH. This was consistent with a study by Schwarz & Elman [31], who found that severity of cardiac illness was predictive of hospital readmission in a population of patients with heart failure. Furthermore, Woolley et al. [26] observed that diagnoses of terminal heart and lung disease were associated with more acute hospital admissions as compared to other diagnoses among hospice patients. Saunders [10] found that in a population of patients with heart failure, hospitalizations for 65% of the patients were cardiac related, which was expected given their diagnosis. The remaining 35% of the hospitalizations were mostly for respiratory-related conditions including pneumonia, pleural effusion, and obstructive pulmonary disease.

Having a poor self-perception of health was also found to be a significant predictor of the occurrence of EDVH. This was consistent with Weaver & Weaver’s [7] findings that inpatients 18+ years of age who identified as having “poor health” were more likely to have a hospitalization than those who identified as having “good health” or “fair health”. Individuals who believed they had poor health may have been more likely to request the use of acute care services. Statistics Canada [32] reports that perceived health is often more effective than clinical measures for predicting help-seeking behaviour and health service use.

According to Weaver & Weaver [7], age and gender were significant predictors of hospitalization, which was inconsistent with our findings. This disparity may have been due to the current study sample’s average age (81.55 years), which was much older than the sample in Weaver & Weaver’s [7] study (47.2 years). Weaver & Weaver [7] also measured age and gender as interaction terms (e.g. 18–39 male, 18–39 female) and found that disparities among genders only existed at younger ages, as both elderly males and females were at significantly increased risk of hospitalizations. This result was consistent with our study.

The non-significant result for ADL decline was surprising given that similar physical limitation measures showed significance in other studies examining EDVH use [4, 7, 31]. As previously mentioned, this may have been due to the relatively older age of our sample as compared to that of Salam-White et al. [4] and Weaver & Weaver [7].

### Strengths and limitations

To our knowledge, this was one of the most comprehensive examinations on the presence of informal caregivers and its association with the occurrence of an EDVH. The use of the interRAI, which is a gold-standard health assessment tool, allowed us to obtain standardized responses and ensures comparability with future research or health initiatives. Additionally, using two years’ worth of hospital-based client data helped ensure a relatively large sample size.

The primary limitation of the study was that data were obtained from only one hospital, which limits generalizability to the entire population. Specifically, given that the data were obtained from only one center, and the participants were on average over 81 years of age, findings may not be representative of all older adults, or those outside of an urban area. Also note that all participants had a recent hospitalization, further limiting generalizability to the entire population. In addition, a positive outcome for acute care visits was defined as having at least one EDVH within 180 days after admission. Alternative models examining the number of EDVH events or their cost may provide additional information.

## Conclusions

Informal caregiver support was not significantly associated with the occurrence of an EDVH in this elderly population receiving home care. However, other key factors were significantly associated with an EDVH, including participants having a poor perception of their health, severe cardiac disorders, and pulmonary diseases. Future research should examine the potential associations between length of hospital stay or quality of life and the presence of an informal caregiver. Should this association prove significant, resources and policies may be improved to further support caregivers, while additional resources could be provided to those who do not have access to an informal caregiver. In general, our work contributes to a growing body of literature that is increasingly concerned with the health of our aging population, and more specifically, health service use by elderly patients, which may have implications for health care providers.

## Abbreviations

CCAC: Central Community Care Access Centre
CI: confidence interval
DAD: Discharge Abstract Database
EDVH: Emergency department visits and hospitalizations
MDS-HC: Minimum Data set – Home Care
NACRS: National Ambulatory Care Reporting System
OR: odds ratio
